# Role of Junctional Adhesion Molecule-C in the Regulation of Inner Endothelial Blood-Retinal Barrier Function

**DOI:** 10.3389/fcell.2021.695657

**Published:** 2021-06-07

**Authors:** Xu Hou, Hong-Jun Du, Jian Zhou, Dan Hu, Yu-Sheng Wang, Xuri Li

**Affiliations:** ^1^Department of Ophthalmology, Eye Institute of Chinese PLA, Xijing Hospital, Fourth Military Medical University, Xi’an, China; ^2^State Key Laboratory of Ophthalmology, Zhongshan Ophthalmic Center, Sun Yat-sen University, Guangzhou, China

**Keywords:** junctional adhesion molecule-C, blood-retinal barrier, retinal capillary endothelial cell, vascular endothelial growth factor, platelet-derived growth factor-C

## Abstract

Although JAM-C is abundantly expressed in the retinae and upregulated in choroidal neovascularization (CNV), it remains thus far poorly understood whether it plays a role in the blood-retinal barrier, which is critical to maintain the normal functions of the eye. Here, we report that JAM-C is highly expressed in retinal capillary endothelial cells (RCECs), and VEGF or PDGF-C treatment induced JAM-C translocation from the cytoplasm to the cytomembrane. Moreover, JAM-C knockdown in RCECs inhibited the adhesion and transmigration of macrophages from wet age-related macular degeneration (wAMD) patients to and through RCECs, whereas JAM-C overexpression in RCECs increased the adhesion and transmigration of macrophages from both wAMD patients and healthy controls. Importantly, the JAM-C overexpression-induced transmigration of macrophages from wAMD patients was abolished by the administration of the protein kinase C (PKC) inhibitor GF109203X. Of note, we found that the serum levels of soluble JAM-C were more than twofold higher in wAMD patients than in healthy controls. Mechanistically, we show that JAM-C overexpression or knockdown in RCECs decreased or increased cytosolic Ca^2+^ concentrations, respectively. Our findings suggest that the dynamic translocation of JAM-C induced by vasoactive molecules might be one of the mechanisms underlying inner endothelial BRB malfunction, and inhibition of JAM-C or PKC in RCECs may help maintain the normal function of the inner BRB. In addition, increased serum soluble JAM-C levels might serve as a molecular marker for wAMD, and modulating JAM-C activity may have potential therapeutic value for the treatment of BRB malfunction-related ocular diseases.

## Introduction

A healthy blood-retinal barrier (BRB) serves to balance the circulation of molecules and to maintain a homeostatic environment for the normal function of the neural retina (Hudson and Campbell, 2019). The inner and outer BRBs are formed by retinal capillary endothelial cells (RCECs) and retinal pigment epithelial cells (RPECs) in collaboration with Bruch’s membrane and the choriocapillaris (Liu and Liu, 2019). Breakdown of the BRB is a common feature of many ocular diseases, such as diabetic retinopathy, wet age-related macular degeneration (wAMD), retinal vein occlusions, uveitis, and other chronic retinal diseases, which, if uncontrolled, can lead to blindness (Klaassen et al., 2013). Three types of junction molecules have been identified in retinal cell junctions: tight junctions (TJs), adherens junctions, and gap junctions (Klaassen et al., 2013). These junctions are dynamic structures (Kowalczyk and Nanes, 2012; Solan and Lampe, 2018; Heinemann and Schuetz, 2019). Junction molecules cycle continuously between the plasma membrane and intracellular compartments. TJs are the apical type of junction and are essential for cell polarity, whereas adherens junctions and gap junctions are organized in a scattered lateral distribution (Garrido-Urbani et al., 2014). TJs are the main junction type in both RCECs and RPECs contributing to BRB function (Cunha-Vaz et al., 2011). However, their composition and architecture are quite different (Dejana, 2004). After the identification of TJ molecules, including zonula occludens (ZO) (Stevenson et al., 1986), occludin (Furuse et al., 1993), and claudins (Furuse et al., 1998), the junctional adhesion molecule (JAM) family was discovered, and their functions in TJ dynamics and leukocyte transmigration were elucidated (Garrido-Urbani et al., 2014).

As a member of the classical JAM family, JAM-C was found to be abundantly expressed in the neural retinae and RPECs (Daniele et al., 2007). In our previous study, JAM-C was detected primarily in the RPEC layer, the inner and outer segments, and the inner plexiform layer of the retina. Meanwhile, JAM-C expression was found to be upregulated in the choroid-RPEC complex with choroidal neovascularization (CNV), whereas no such change was found in the neural retina. JAM-C blockade has been shown to suppress CNV formation by inhibiting macrophage transmigration and by reducing RPEC malfunction (Hou et al., 2012). Different from other members of the JAM family, JAM-C has been shown to regulate leukocytes to exit the abluminal compartment by reverse transmigration (Woodfin et al., 2011). However, it remains thus far unclear whether JAM-C plays a role in the regulation of inner endothelial BRB function, particularly, in relationship to ocular diseases.

In this study, we examined JAM-C expression in cultured human RCECs, and observed that JAM-C translocated from the cytoplasm to cytomembrane after treatment with vascular endothelial growth factor (VEGF) or platelet-derived growth factor-C (PDGF-C), two growth factors known to be critical for the function and morphology of the vascular system. Moreover, cytosolic Ca^2+^ concentrations in RCECs and the infiltration ability of macrophages from wAMD patients and healthy controls were investigated to verify the potential effect of JAM-C on the inner BRB. Importantly, we found that serum soluble JAM-C levels increased in wAMD patients, indicating a potential possibility of using soluble JAM-C as a biomarker for wAMD.

## Materials and Methods

### Human RCECs and Macrophages

Primary human RCECs were purchased from the Type Culture Collection of the Chinese Academy of Sciences (Shanghai, China) and were cultured in endothelial cell medium (ScienCell, Shanghai, China) containing 10% fetal bovine serum (FBS) at 37°C in a 5% CO_2_ incubator. Cells of the third to fifth passages were used in all experiments.

All patients included in this study signed informed consent and the study was approved by the ethical and academic boards of Xijing Hospital of the Fourth Military Medical University. All procedures used conformed to the tenets of the Declaration of Helsinki. Peripheral blood monocytes from age- and gender-matched healthy adult donors and wAMD patients were isolated using a published method (Hou et al., 2012). Briefly, peripheral blood monocytes were isolated from leukopheresed buffy coat fractions after density gradient centrifugation using an aqueous medium (Amersham Biosciences, Piscataway, NJ) according to the manufacturer’s protocol. Anti-human CD14 microbeads (Miltenyi Biotec, Cambridge, MA) were used for positive selection/purification of monocytes using a magnetic cell separation instrument (AutoMACS; Miltenyi Biotec, Gaithersburg, MD). The purity of the CD14^+^ cell population was higher than 90%. Complete medium (Biosource, Rockville, MD) consisting of RPMI 1640 supplemented with penicillin (100 U/mL), streptomycin (100 U/mL), L-glutamine (2 mM), and 10% FBS was used for monocyte culture. The cells were cultured in the presence of macrophage-colony stimulating factor (PeproTech, Rocky Hill, NJ) for 6 days for differentiation into macrophages.

### Immunofluorescence

Human RCECs were cultured on chamber slides in 4-well plates to approximately 90% confluence in serum-free medium overnight before experiments. The cells were then treated with PDGF-C (125 ng/mL, Sino Biological Inc., Beijing, China) or VEGF (10 ng/mL, Sino Biological Inc., Beijing, China) for 1 hr. The slides were then washed, fixed in 4% paraformaldehyde for 10 min, and permeabilized with 0.1% Triton X-100 in phosphate-buffered saline (PBS) for 5 min. The slides were blocked with 1% bovine serum albumin and 5% goat serum in PBS at room temperature for 1 hr. After incubation with rat anti-JAM-C (CRAM-18 F26) and rabbit anti-ZO1 (ab59720) (Abcam, Shanghai, China) antibodies and secondary antibodies (Alexa Fluor 488 and Alexa Fluor 594, Yeasen, Shanghai, China). The slides were washed and mounted in medium (DAPI Fluoromount G; SouthernBiotech, Birmingham, AL). A negative control staining followed the same protocol except that the anti-JAM-C or anti-ZO1 antibodies. Fluorescence images were captured using a laser scanning microscope (Leica Microsystems, Wetzlar, Germany).

### Western Blot

Human RCECs in 6-well plates were treated with VEGF (10 ng/mL) or PDGF-C (125, 250 ng/mL) for 1 hr. After washing with PBS, the cells were lysed using a protein extraction kit (Beyotime, Shanghai, China). Equal amounts of protein were electrophoresed in 10% sodium dodecyl sulfate-polyacrylamide gels and transferred onto polyvinylidene difluoride membranes. The membranes were probed with a rabbit anti-JAM-C antibody (Abcam, Shanghai, China). An anti-rabbit horseradish peroxidase-conjugated antibody (Pierce, Rockford, IL) was used as a secondary antibody. An enhanced chemiluminescence kit (SuperSignal Pico ECL; Pierce, Rockford, IL) was used to detect the signal using an imaging system (LAS-3000 Imager; Fujifilm, Tokyo, Japan). Densitometric quantification of the bands was performed using the ImageJ program (NIH, United States).

### Lentiviral Vectors and Cell Transfection

For JAM-C knockdown, the lentiviral vector U6-MCS-Ubiquitin-Cherry-IRES-puromycin (GV298) encoding a JAM-C-RNAi sequence and the control lentiviral vector were purchased from Genechem Technology (Shanghai, China). Three different targeting sequences for JAM-C knockdown were used: 5′-TGAACATTGGCGGAATTAT-3′, 5′-AATCCCAGATTTCGCAATT-3′, and 5′-GCAGGAG ATGGAAGTCTAT-3′. Knockdown efficiencies were evaluated by quantitative reverse-transcription (RT-q) PCR and Western blot. The lentiviral vector with the highest knockdown efficiency was used for experiments.

For JAM-C overexpression, the lentiviral vector Ubi-MCS-3FLAG-SV40-puromycin (GV341) encoding human JAM-C and the control lentiviral vector (GV341-JAM-C) were purchased from Genechem Technology. Human RCECs were infected with GV341-JAM-C or control vector. JAM-C expression was assessed by RT-qPCR and Western blot.

### RT-qPCR

Total cellular RNA was isolated using TRIzol reagent (Takara, Dalian, China). A One-Step RT-PCR kit from Takara was used following the manufacturer’s instruction. The forward and reverse primers used for *JAM-C* were: 5′-GAGACTCAGCCCTTTATCGC-3′ and 5′-CCTTCGGCACTCTACAGACA-3′, and for ACTB: 5′- TGGACTTCGAGCAAGAGATG-3′ and 5′- GAAGGA AGGCTGGAAGAGTG-3′. SYBR Green PCR Master Mix (Applied Biosystems, Foster City, CA) was used for qPCRs. PCR products were analyzed by electrophoresis (Bio-Rad, Hercules, CA). mRNA levels were normalized to β-actin mRNA. Each experiment was repeated three times.

### Flow Cytometry

Human RCECs were treated with VEGF (10 ng/mL) or PDGF-C (125 ng/mL) for 1 hr. After treatment with Fluo-3 (Invitrogen, Carlsbad, CA) in 1% working solution at 37°C for 30 min, the cells were washed three times with Ca^2+^-free PBS and resuspended in PBS at 1 × 10^6^ cells/mL. Cytosolic Ca^2+^ concentrations were detected by flow cytometry at an excitation wavelength of 488 nm and an emission wavelength of 530 nm (BD Biosciences, San Jose, CA). Cells not treated with Fluo-3 served as controls.

### Enzyme-Linked Immunosorbent Assay (ELISA)

Soluble JAM-C protein levels in the serum of wAMD patients (*n* = 10) and healthy controls were determined using a human JAM-C ELISA kit from USCN Life Science Inc. (Wuhan, China). The absorbance at 450 nm was measured using an ELX-800 Microplate Reader (BioTek Instruments Inc., Winooski, VT).

### Macrophage Adhesion and Transwell Migration Assays

Macrophage adhesion assays were performed as described previously (Zhou et al., 2018). Briefly, human RCECs were cultured to confluence in 96-well plates. After treatment with lipopolysaccharide (LPS) for 4 h, macrophages from wAMD patients were added into 96-well plates (10^5^/well) containing the RCEC monolayers and incubated for 1 h. After washing with PBS, the cells were imaged using an inverted microscope and adhered macrophages were counted in five random fields.

Macrophage Transwell migration assays were performed as described previously (Zhou et al., 2018). Briefly, human RCECs were cultured to confluence on the upper layer of a permeable membrane coated with collagen (Sigma-Aldrich, St. Louis, MO). Then, 600 μL of RPMI medium containing 50 ng/mL monocyte chemotactic protein-1 (MCP-1) (R&D Systems, Minneapolis, MN) was added to the lower chamber, and LPS-treated macrophages (4 × 10^5^/well) were placed on the upper layer and allowed to migrate for 4 h. The macrophages that had migrated across the membrane to the lower chamber were stained with a 0.5% solution of gentian violet for 5 min and were counted in 5 random fields under a microscope. For protein kinase C (PKC) inhibition, confluent RCECs were pretreated with 1 μM GF109203X (Merck-Calbiochem, Darmstadt, Germany) for 2 h before MCP-1 was added to the lower chamber.

### Statistical Analysis

Data are represented as the mean ± standard error of the mean and were analyzed using analysis of variance assuming equal variances. *P* < 0.05 was considered statistically significant. All experiments were conducted in triplicate.

## Results

### JAM-C Is Expressed in Human RCECs

**FIGURE 1:**
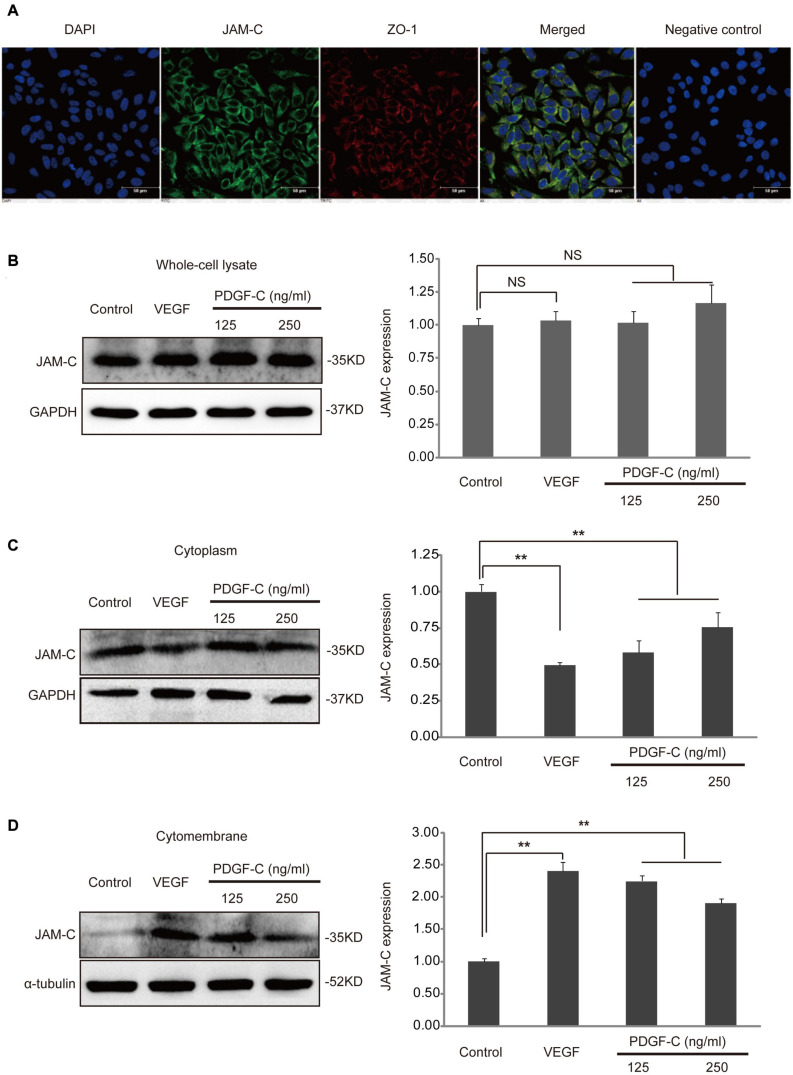
JAM-C expression and translocation in human RCECs. **(A)** JAM-C expression in cultured human RCECs is shown by immunofluorescence staining (blue: DAPI, green: JAM-C, red: ZO-1, scale bar: 50 μm). JAM-C co-localized with ZO-1 in RCECs. A negative control staining without the primary antibody against JAM-C or ZO-1 showed no staining. **(B)** Cultured human RCECs were treated with VEGF (10 ng/mL) or PDGF-C (125, 250 ng/mL) for 1 h. JAM-C protein in the whole-cell lysate was similar as shown by Western blot. **(C)** In the cytoplasmic fraction, JAM-C protein levels decreased after VEGF or PDGF-C protein treatment. **(D)** In the cytomembrane fraction, JAM-C protein levels increased after VEGF or PDGF-C treatment. The dynamic changes in JAM-C translocation induced by PDGF-C were dose-dependent. **P < 0.01, NS, not significant.

To investigate the potential role of JAM-C in RCECs, we checked whether these cells express JAM-C. Double immunostaining showed that JAM-C was expressed in RCECs and co-localized with ZO-1 (Figure 1A), an important TJ molecule critical for normal inner BRB function (Deissler et al., 2020). These results suggested a potential role of JAM-C in human RCECs.

### VEGF and PDGF-C Induce Translocation of JAM-C

Under pathological conditions, such as inflammation, ischemia, or neovascularization, TJ molecules function to adjust the endothelial barrier status (Campbell and Humphries, 2012). Thus, we used VEGF and PDGF-C, two important angiogenic factors (Hou et al., 2010), to stimulate RCECs and investigated JAM-C expression in different cellular compartments, including whole-cell lysate, cytomembrane, and cytoplasm. JAM-C expression was detected in the whole-cell lysate, and JAM-C protein levels were similar after VEGF or PDGF-C treatment (Figure 1B). However, in the cytoplasm fraction, JAM-C protein levels decreased after VEGF or PDGF-C treatment (*P* < 0.01, Figure 1C). In contrast, in the cytomembrane fraction, JAM-C protein levels increased after VEGF or PDGF-C treatment, with (*P* < 0.01, Figure 1D). Together, these data demonstrated that VEGF and PDGF-C stimulation induces translocation of JAM-C from cytoplasm to cytomembrane, indicating a potential role of JAM-C in VEGF- or PDGF-C-related effects.

### JAM-C Knockdown and Overexpression in RCECs

**FIGURE 2:**
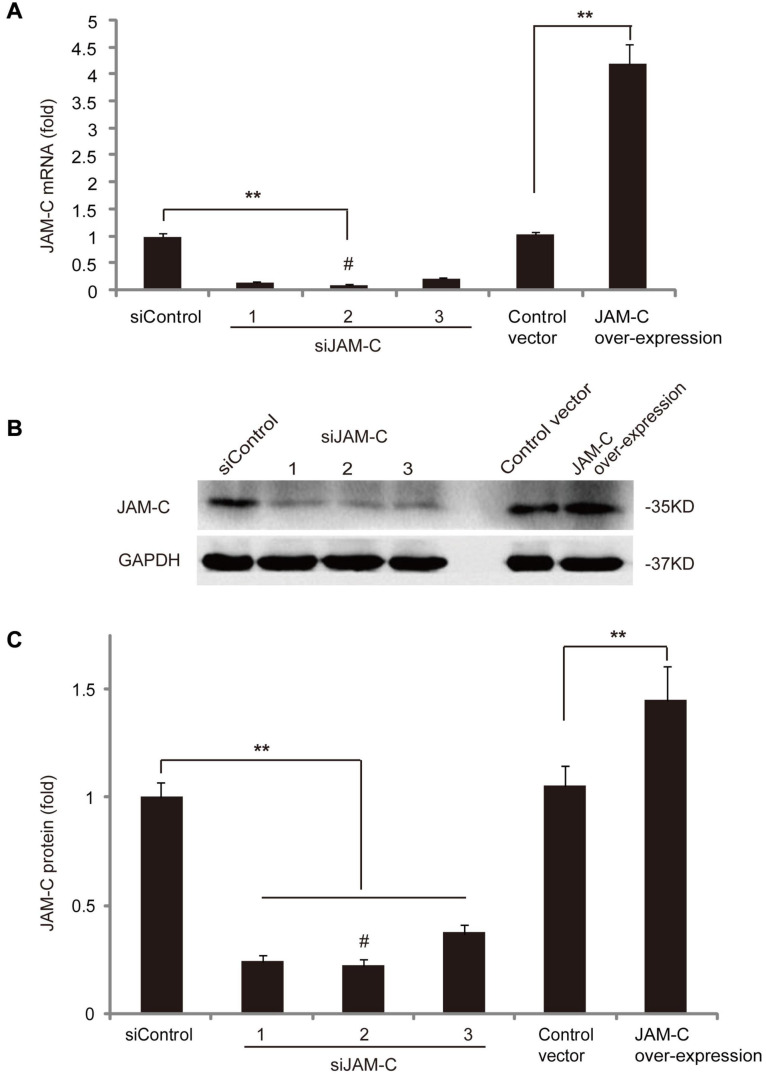
JAM-C overexpression and knockdown in human RCECs. **(A)** Three different siRNAs for JAM-C knockdown were cloned into lentiviral vectors and transfected into RCECs. Knockdown efficiency was verified by RT-qPCR. The second siRNA construct most effectively reduced JAM-C mRNA expression to about 12%. For gain-of-function analysis, human *JAM-C* was cloned into a lentiviral vector and transfected into RCECs. RT-qPCR showed an approximately 4.3-fold increase of JAM-C transcripts. **(B,C)** For loss-of-function analysis, Western blot showed that the second siRNA construct was the most efficient and reduced JAM-C protein level to about 25%. For gain-of-function analysis, Western blot showed an approximately 1.5-fold increase of JAM-C protein in RCECs overexpressing human *JAM-C*. **P < 0.01. ^#^: Used for subsequent experiments.

To evaluate the role of JAM-C in RCECs, we conducted loss- and gain-of-function analyses using JAM-C knockdown and overexpression, respectively. For JAM-C knockdown, three different siRNAs were cloned into a lentiviral vector and transfected into RCECs. Knockdown efficiency at the mRNA level was verified by RT-qPCR, which showed that the second siRNA construct reduced JAM-C mRNA expression to about 12% of the basal level (Figure 2A). Western blot analysis also showed that the second siRNA was the most effective and the reduced JAM-C protein level to about 25% of the basal level (Figures 2B,C). Thus, RCECs transfected with the second siRNA construct were used in subsequent loss-of-function experiments.

For gain of function assay, a lentiviral vector expressing human *JAM-C* was transfected into RCECs. RT-qPCR revealed an approximately 4.3-fold increase in JAM-C mRNA expression (Figure 2A), and Western blot showed an approximately 1.5-fold increase in the JAM-C protein level (Figures 2B,C). RCECs overexpressing JAM-C were used in subsequent experiments.

### JAM-C Decreases Cytosolic Ca^2+^ Concentrations in RCECs

**FIGURE 3:**
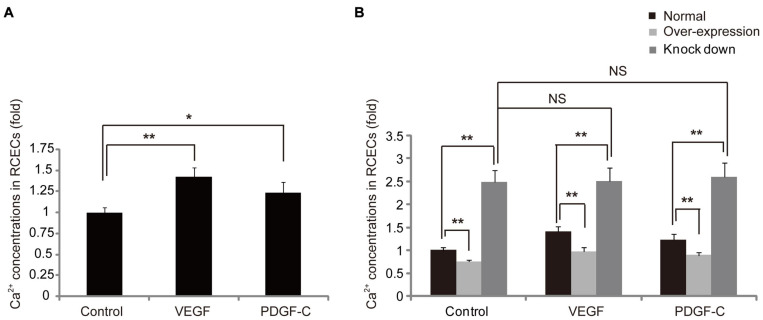
JAM-C decreases cytosolic Ca^2+^ concentrations in human RCECs. Cultured human RCECs were treated with VEGF (10 ng/mL) or PDGF-C (125 ng/mL) for 1 h. Cytosolic Ca^2+^ concentrations were detected by flow cytometry. **(A)** VEGF and PDGF-C increased cytosolic Ca^2+^ concentrations in RCECs. **(B)** JAM-C overexpression decreased cytosolic Ca^2+^ concentrations, whereas JAM-C knockdown increased Ca^2+^ concentrations in RCECs. VEGF and PDGF-C did not increase calcium influx in RCECs after JAM-C knockdown. *P < 0.05, **P < 0.01, NS, not significant.

It is known that vasoactive molecules stimulate their receptors on endothelial cells to initiate signaling that increases cytosolic Ca^2+^, and Ca^2+^ activation disrupts TJs (Vandenbroucke et al., 2008). Therefore, we used VEGF and PDGF-C to stimulate RCECs and found that cytosolic Ca^2+^ increased in endothelial cells (Figure 3A). We further investigated the potential role of JAM-C in the regulation of Ca^2+^ activation. Regardless of VEGF or PDGF-C treatment, overexpression and knockdown of JAM-C in RCECs decreased and markedly increased cytosolic Ca^2+^ concentrations, respectively (Figure 3B), indicating that increased endothelial Ca^2+^ levels could be reversed through JAM-C overexpression to “strengthen” TJs. However, VEGF and PDGF-C treatment did not increase Ca^2+^ influx in endothelial cells after JAM-C knockdown, suggesting that Ca^2+^ activation induced by VEGF and PDGF-C may require JAM-C, an essential molecule of TJs.

### Inhibition of JAM-C in RCECs Decreases Macrophage Adhesion to RCECs

**FIGURE 4:**
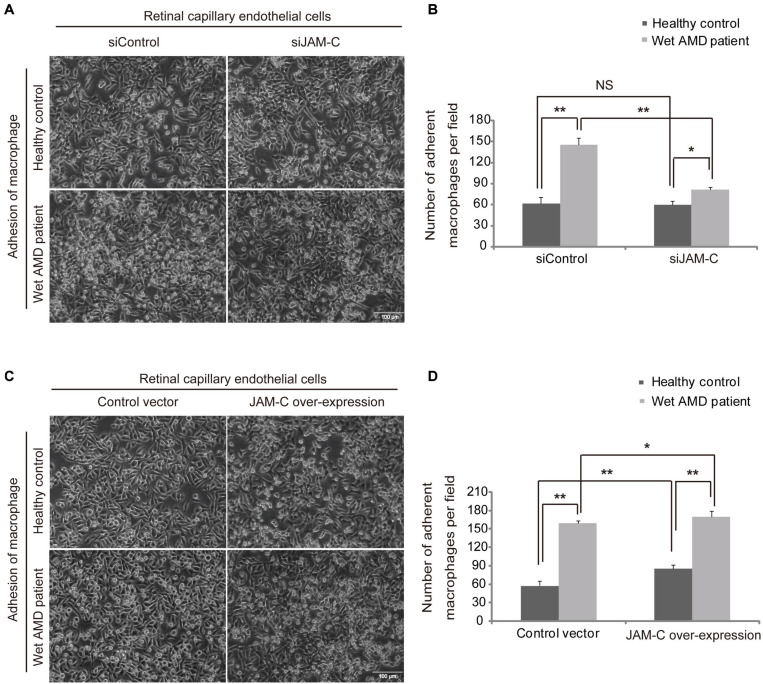
JAM-C regulates macrophage adhesion to human RCECs. Macrophages from wAMD patients or healthy controls were added to culture plate wells containing a confluent monolayer of human RCECs, and the plates were incubated for 1 h. After washing the wells with PBS, adherent cells in five random fields were counted. Adherent macrophages were identified based on the cell size and morphology. **(A,B)** JAM-C knockdown in RCECs inhibits the adhesion of macrophages (small and round in shape) from wAMD patients to RCECs, but not those from healthy controls. The adhesion ability of macrophages from wAMD patients in both the siControl and siJAM-C groups increased compared to that of macrophages from healthy controls. **(C,D)** JAM-C overexpression increased the adhesion of macrophages from both wAMD patients and healthy controls. The adhesion ability of macrophages from wAMD patients in both the control and JAM-C overexpression groups increased compared to those from healthy controls. Scale bar: 100 μm, *P < 0.05, **P < 0.01, NS, not significant.

Macrophage adhesion to vascular endothelial cells is critical for their activation and infiltration in immune or inflammatory diseases (Futosi et al., 2013). To investigate the potential role of JAM-C in the regulation of inner BRB function, we investigated the adhesion ability of macrophages derived from wAMD patients or healthy controls to RCECs after JAM-C knockdown (siJAM-C). We found that the mean numbers of macrophages from healthy controls adhered to RCECs treated with siJAM-C were 59.4 ± 81.2 per field in the siJAM-C group and were 61.6 ± 9.3 per field in the siControl group with no statistical difference (Figures 4A,B). The mean numbers of macrophages from wAMD patients adhered to RCECs treated with siJAM-C were 81.2 ± 3.7 in the siJAM-C group and were 145.8 ± 9.3 in the siControl group (*P* < 0.01, Figures 4A,B), demonstrating that JAM-C knockdown inhibited the adhesion of macrophages from wAMD patients, but not of those from healthy controls. Moreover, the adhesion ability of macrophages from wAMD patients in both the siJAM-C and siControl groups increased compared to that of macrophages from healthy controls (Figure 4B). These data suggest that the enhanced adhesion of macrophages in wAMD could be reduced by targeting JAM-C without affecting healthy macrophages.

### Overexpression of JAM-C in RCECs Increases Macrophage Adhesion to Them

Using RCECs overexpressing JAM-C, we found that the mean numbers of macrophages from healthy controls adhered to RCECs were 57.8 ± 8.0 in the control group and were 85.6 ± 6.5 in the JAM-C overexpression group (*P* < 0.01, Figures 4C,D). The mean numbers of macrophages from wAMD patients adhered to RCECs were 159.6 ± 4.2 in the control group and were 170.2 ± 9.0 in the JAM-C overexpression group (*P* < 0.05, Figures 4C,D). These findings showed that JAM-C overexpression increased the adhesion of macrophages from both wAMD patients and healthy controls. In addition, the adhesion ability of macrophages from wAMD patients in both the control and JAM-C overexpression groups increased compared to that of macrophages from healthy controls (Figure 4D). Thus, increased JAM-C expression in vascular endothelial cells may impair inner BRB function by facilitating macrophage adhesion.

### JAM-C Knockdown/Overexpression in RCECs Decreases/Increases Macrophage Transmigration Through RCECs

**FIGURE 5:**
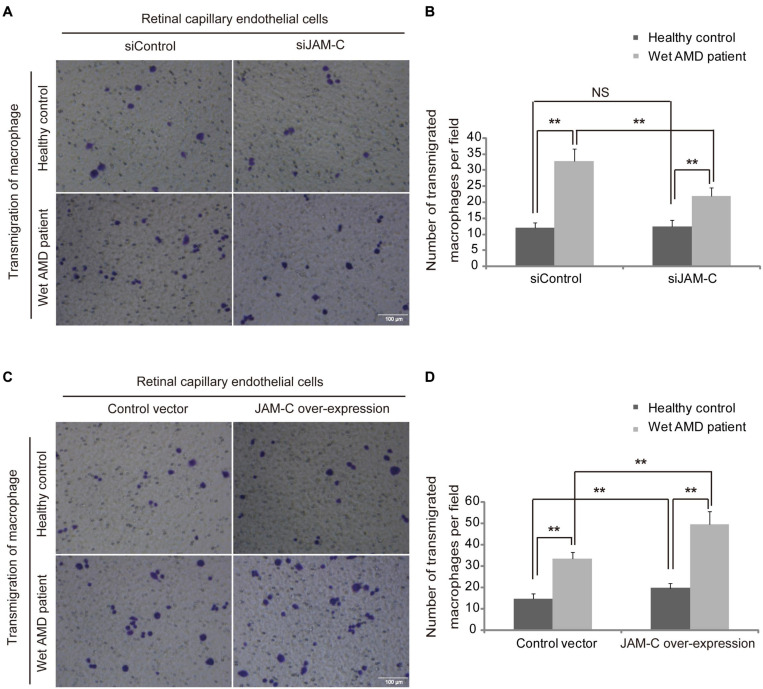
JAM-C regulates macrophage transmigration through human RCECs. RCECs were cultured to confluence on the upper layer of a permeable membrane, and then macrophages were placed on the upper chamber and allowed to migrate through the RCEC monolayer to the lower chamber for 4 hr. Transmigrated macrophages were counted in five random fields. **(A,B)** JAM-C knockdown inhibited the transmigration of macrophages from wAMD patients, but not those from healthy controls. The transmigration of macrophages from wAMD patients in both the siControl and siJAM-C groups increased compared to those from healthy controls. **(C,D)** JAM-C overexpression increased the transmigration of macrophages from both wAMD patients and healthy controls. The transmigration of macrophages from wAMD patients in both the control and JAM-C overexpression groups increased compared to those from healthy controls. Scale bar: 100 μm, **P < 0.01, NS, not significant.

JAM-C-induced increase of macrophage transmigration through retinal pigment epithelium, the structure of the outer BRB, contributes to CNV (Hou et al., 2012). To verify the potential role of JAM-C in the regulation of macrophage transmigration through the inner BRB, we investigated the transmigration ability of macrophages from wAMD patients and healthy controls through monolayers of RCECs after JAM-C knockdown. The mean numbers of macrophages from healthy controls transmigrated through an RCEC monolayer were 12 ± 1.6 per field in the siControl group and were 12.4 ± 2.1 per field in the siJAM-C group (Figures 5A,B) with no statistical difference. The mean numbers of macrophages from wAMD patients transmigrated through an RCEC monolayer were 32.8 ± 3.7 in the siControl group and were 21.8 ± 2.6 in the siJAM-C group (*P* < 0.01, Figures 5A,B), indicating that JAM-C knockdown inhibited the transmigration of macrophages from wAMD patients, but not of those from healthy controls. In addition, the transmigration ability of macrophages from wAMD patients in both the siControl and siJAM-C groups increased compared to that of macrophages from healthy controls (Figure 5B). These data thus suggest that JAM-C targeting to inhibit macrophage transmigration may be useful for wAMD treatment without affecting healthy macrophages.

Using RCECs overexpressing JAM-C, we found that the mean numbers of macrophages from healthy controls transmigrated through an RCEC monolayer were 14.6 ± 2.4 in the control group and were 19.6 ± 2.3 in the JAM-C overexpression group (*P* < 0.01, Figures 5C,D). The mean numbers of macrophages from wAMD patients transmigrated through an RCEC monolayer were 33.4 ± 3.1 in the control group and were 49.4 ± 6.2 in the JAM-C overexpression group (*P* < 0.01, Figures 5C,D). These findings indicated that JAM-C overexpression increased the transmigration of macrophages from both wAMD patients and healthy controls. The transmigration ability of macrophages from wAMD patients in both the control and JAM-C overexpression groups increased compared to that of macrophages from healthy controls (Figure 5D). These findings indicate that increased JAM-C expression may exacerbate macrophage infiltration and lead to a breakdown of the inner BRB in wAMD.

### Inhibition of PKC in RCECs Decreases Transmigration of Macrophages From wAMD Patients

**FIGURE 6:**
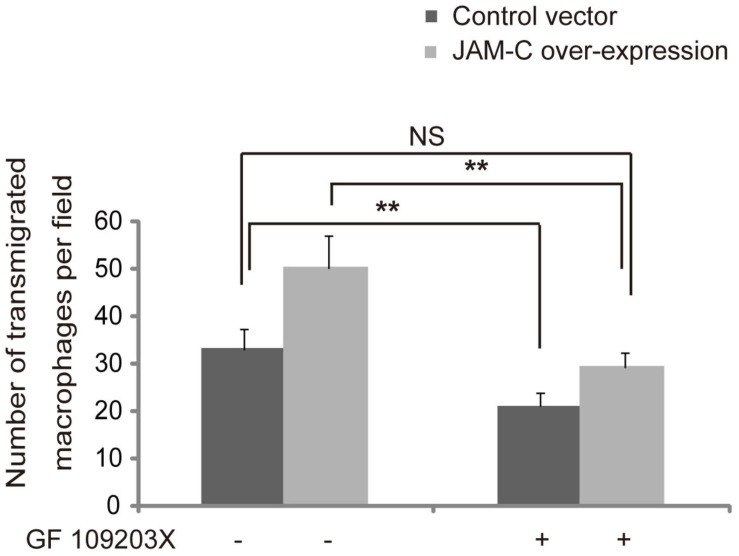
Inhibition of PKC decreases the transmigration of macrophages from wAMD patients. An RCEC monolayer was pretreated with 1 μM of GF109203X for 2 hr. Macrophages from wAMD patients were then added and allowed to migrate for 4 h. Transmigrated macrophages were stained with a 0.5% gentian violet for 5 min and counted in five random fields. After GF109203X treatment, macrophage transmigration decreased in both JAM-C overexpressing and control RCECs. Compared with the control group without GF109203X treatment, JAM-C overexpressing RCECs treated with GF109203X did not induce any increase in macrophage transmigration. **P < 0.01, NS, not significant.

It has been shown that the translocation of JAMs is, at least in part, regulated by the PKC pathway (Sarelius and Glading, 2015). To investigate whether the increased transmigration of macrophages from wAMD patients promoted by JAM-C could be inhibited by PKC suppression, we treated RCECs with the PKC inhibitor GF109203X, and found that the transmigration of macrophages from wAMD patients decreased for both JAM-C-overexpressing and control RCECs after GF109203X treatment (*P* < 0.01, Figure 6). Compared with the control cells without GF109203X treatment, JAM-C-overexpressing RCECs treated with GF109203X did not increase macrophage transmigration (Figure 6). Thus, the increased macrophage transmigration induced by JAM-C overexpression in RCECs was suppressed by PKC inhibition, suggesting that JAM-C regulates inflammatory cell infiltration at least in part via the PKC pathway.

### Soluble JAM-C Levels Are Elevated in the Sera of wAMD Patients

Increased soluble JAM-C levels have been shown to be related to vascular inflammatory diseases (Manetti et al., 2013). To investigate whether soluble JAM-C is involved in wAMD, we measured soluble JAM-C levels in the sera of wAMD patients (Table 1) and healthy controls by ELISA. We found that the mean concentration of soluble JAM-C in the control group was 475.8 ± 77.9 pg/mL, and was 1223.3 ± 115.2 pg/mL in the sera of wAMD patients (*P* < 0.05, *n* = 10). These findings indicate that increased soluble JAM-C levels in the serum might be able to serve as a biomarker for wAMD.

**TABLE 1:** Clinical information of all patients and soluble JAM-C levels.

**Patient**	**Age (years)**	**Gender (Male/Female)**	**Soluble JAM-C (pg/mL)**
1	69	M	1393.9
2	55	M	1189.3
3	71	M	1006.7
4	66	F	1286.2
5	65	F	1085.3
6	77	M	1228.7
7	82	F	1259.7
8	63	F	1193.2
9	72	F	1353.4
10	76	M	1236.5
Average	69.6 ± 7.8		1223.3 ± 115.2

## Discussion

JAMs are a family of transmembrane proteins that play vital roles in the regulation of TJ dynamics. The roles of JAM-C in the development of the retinal structure (Daniele et al., 2007; Li et al., 2018), revascularization of the hypoxic retina (Economopoulou et al., 2015), RPEC BRB formation (Economopoulou et al., 2009) and CNV (Hou et al., 2012) suggest its potential as a therapeutic target in the management of various retinal diseases. In this work, we found that JAM-C is expressed in RCECs and regulates the inner endothelial BRB function through its dynamic changes in cellular distribution. Increased JAM-C expression in human RCECs may lead to loss of retinal homeostasis by promoting macrophage infiltration, which can be inhibited by PKC inhibitor treatment.

The translocation of JAM-C upon stimulation with vasoactive molecules, such as VEGF, in macrovascular endothelial cells is different from that in microvascular endothelial cells. In the former, JAM-C is constitutively located at the interendothelial contacts and its localization is not affected by stimulation with vasoactive molecules, whereas in the latter, JAM-C is recruited from the cytoplasm to the cell-cell contacts after vasoactive molecule stimulation (Orlova et al., 2006). Accordingly, we found that RCECs showed a similar pattern as other microvascular endothelial cells, and total JAM-C did not change upon stimulation with VEGF or PDGF-C. Additionally, we also found that PDGF-C-induced JAM-C translocation in RCECs is dose-dependent. Our findings suggest that vasoactive molecules might serve as a switch to activate JAM-C in retinal diseases.

As an essential second messenger of endothelial cells, calcium plays a critical role in regulating barrier function and inflammation. And increased cytosolic Ca^2+^ levels can induce tight junction disassembly, increased permeability (Filippini et al., 2019; Dalal et al., 2020), and angiogenesis (Wong and Yao, 2011). VEGF has been shown to induce cytosolic Ca^2+^ elevation in retinal endothelial cells (Hiroishi et al., 2007). PDGF family molecules are potent angiogenic and survival factors (Kumar and Li, 2018). PDGF-BB can affect the integrity of the retinal microvasculature by modulating the intracellular calcium homeostasis of pericytes (Knorr et al., 1995). In this work, we showed that PDGF-C induced an increase of cytosolic Ca^2+^ in human RCECs, supporting a role of PDGF-C in pathological angiogenesis reported previously (Hou et al., 2010). Overexpression of JAM-C in RCECs decreased cytosolic Ca^2+^ levels regardless of PDGF-C treatment. However, after JAM-C knockdown, cytosolic Ca^2+^ markedly increased and treatment with vasoactive molecules did not further increase calcium influx. These data indicate that JAM-C knockdown may increase cytosolic Ca^2+^ more effectively than vasoactive molecules. Collectively, our data suggest that JAM-C is responsible for the maintenance of a dynamic Ca^2+^ balance, which may play an essential role in RCEC barrier function. Currently, how PDGF-C and JAM-C affect cytosolic Ca^2+^ is unclear. VEGF has been shown to induce entry of calcium into endothelial cells via multiple mechanisms, including by regulating calcium ions channel protein (Faehling et al., 2001; Kim et al., 2008; Li et al., 2015). PDGF-C could regulate cytosolic Ca^2+^ level by affecting calcium ions channel protein as well, which remains to be verified. It has also been shown that JAM-A malfunction reduces ATP levels in the mitochondrion and leads to Ca^2+^ overload in sperm (Aravindan et al., 2012). In this work, we found Ca^2+^ accumulation in RCECs after JAM-C knockdown, suggesting that JAM-C might use a similar mechanism to regulate Ca^2+^ in retinal vascular endothelial cells. However, further studies are needed to address this.

Besides its expression in the endothelial cells of TJs, JAM-C exists in a soluble form in human serum and the supernatant of cultured human microvascular endothelial cells (Rabquer et al., 2010). It has been suggested that soluble JAM-C mediates pathological angiogenesis (Rabquer et al., 2010). Serum soluble JAM-C levels increase in sepsis, and blockade of JAM-C can reduce the number of pro-inflammatory neutrophils (Hirano et al., 2018). We found that the mean serum soluble JAM-C concentration in wAMD patients was more than twofold of that of healthy controls. These data are consistent with the finding that increased soluble JAM-C levels correlate with vascular damage (Manetti et al., 2013). Thus, soluble JAM-C levels might be able to serve as a biomarker for pathological neovascular ocular diseases, such as wAMD.

JAM-C has been shown to play a critical role in endothelial permeability and neutrophil transmigration (Hu et al., 2020). Targeting JAM-C may have therapeutic usage in suppressing inflammation (Palmer et al., 2007; Shagdarsuren et al., 2009; Hirano et al., 2018), angiogenesis, and tumor growth (Lamagna et al., 2005) by inhibiting the infiltration of macrophages. Recruited macrophages outside or inside of the BRBs can profoundly influence retinal homeostasis (McMenamin et al., 2019). Both JAM-C and macrophage-1 antigen (Mac1) are expressed in leukocytes, and endothelial cells bind to their ligands for macrophage transmigration during inflammation and angiogenesis (Arrate et al., 2001; Bradfield et al., 2007). Previously, we found that JAM-C blockade inhibits macrophage transmigration to maintain normal RPEC barrier function (Hou et al., 2012). To investigate the role of JAM-C in the regulation of inner BRB function, we overexpressed JAM-C in cultured RCECs and found that the adhesion and transmigration of macrophages were increased. However, knockdown of JAM-C in RCECs did not significantly inhibit the adhesion and transmigration of macrophages from healthy controls, suggesting that under normal conditions, JAM-C in the inner BRB cells may not evidently affect TJ homeostasis. We subsequently investigated macrophages from wAMD patients and found marked increase of adhesion and transmigration of them. Moreover, JAM-C knockdown in RCECs suppressed the adhesion and transmigration of macrophages from wAMD patients. PKCs are serine/threonine kinases (Newton and Johnson, 1998), and JAM phosphorylation by PKC may affect its function and localization (Ozaki et al., 2000). We also found that the increased transmigration of macrophages from wAMD patients could be blocked by pretreatment of RCECs with GF109203X, a PKC inhibitor, indicating that PKC may be involved in the regulation of macrophage transmigration.

In conclusion, our data demonstrated that the dynamic translocation of JAM-C in RCECs and the activation of macrophages induced by vasoactive molecules affect inner endothelial BRB function, and JAM-C upregulation may contribute to the development of wAMD. Although further studies are required to fully clarify the role of JAM-C in ocular pathologies, our findings on the role of JAM-C in the regulation of inner endothelial BRB function may provide a potential therapeutic target for the treatment of BRB malfunction-related ocular diseases.

## Data Availability Statement

The original contributions presented in the study are included in the article/supplementary material, further inquiries can be directed to the corresponding author/s.

## Ethics Statement

The studies involving human participants were reviewed and approved by the ethical and academic boards of Xijing Hospital of the Fourth Military Medical University. The patients/participants provided their written informed consent to participate in this study. Written informed consent was obtained from the individual(s) for the publication of any potentially identifiable images or data included in this article.

## Author Contributions

XH and XL designed the research and wrote the manuscript. XH, H-JD, JZ, DH, and Y-SW performed the experiments. XH, DH, and XL analyzed the data. All authors contributed to the article and approved the submitted version.

## Conflict of Interest

The authors declare that the research was conducted in the absence of any commercial or financial relationships that could be construed as a potential conflict of interest.
